# The Effect of Statins and Other Cardiovascular Medication on Ischemia-Reperfusion Damage in a Human DIEP Flap Model: Theoretical and Epidemiological Considerations

**DOI:** 10.1155/2012/504081

**Published:** 2012-05-09

**Authors:** M. G. W. van den Heuvel, A. Bast, A. W. Ambergen, R. R. W. J. van der Hulst

**Affiliations:** ^1^Department of Plastic, Reconstructive and Hand Surgery, Maastricht University Medical Centre, P.O. Box 5800, 6202 AZ, Maastricht, The Netherlands; ^2^Department of Pharmacology and Toxicology, Maastricht University Medical Centre, P.O. Box 616, 6200 MD, Maastricht, The Netherlands; ^3^Department of Methodology and Statistics, Maastricht University Medical Centre, P.O. Box 616, 6200 MD, Maastricht, The Netherlands

## Abstract

*Background*. Statins and other cardiovascular medication possess antioxidant capacity. It was examined whether chronic use of these medications protects from the development of ischemia-reperfusion (I/R) related complications after DIEP (Deep Inferior Epigastric Perforator Free Flap) surgery. This paper contains a literature study on the antioxidant working mechanisms of these drugs. *Methods*. Medical information of 134 DIEP patients (173 flaps) was studied from their medical files. Patient and operative characteristics were registered, as well as I/R related complications. *Results*. Of the group that didnot use statins, 16.3% developed complications versus 30.8% amongst patients that did use these drugs (*P* = 0.29). Amongst patients that chronically use other cardiovascular medication, 26.8% developed I/R related complications versus 14.4% of the patients without medication (*P* = 0.10). *Conclusions*. Chronic use of statins or other cardiovascular medication didnot decrease the occurrence of I/R related complications after DIEP surgery. Therefore, research should be aimed at evaluating short-term pre-treatment with statins.

## 1. Introduction

Neutrophil influx and the formation of reactive oxygen species (ROS) play an important role in the initiation of ischemia-reperfusion (I/R) damage. An early survey of registered medication showed that many drugs possess antioxidant capacity, which might contribute to their pharmacological action [1]. Statins (3-hydroxy-3-methylglutaryl coenzyme A reductase inhibitors) are inhibitors of sterol synthesis [2]. They block the conversion of HMG-CoA to mevalonate, which is the rate-determining step in cholesterol production [3]. Statins display a pleiotropic activity including antiatherosclerotic, antithrombotic, but also anti-inflammatory and neuroprotective effects [2, 4, 5]. They also exert antioxidant effects [4, 6]. All these effects seem to be independent of their cholesterol-lowering effect [7, 8]. Animal studies have shown a protective effect by administration of statins previously to I/R. They demonstrated that pre-treatment with statins for several days, reduces myocardial injury after I/R [8–11]. Renal function and survival after renal I/R were improved after short-term statin pre-treatment in rats [12, 13]. Improved graft function and decreased renal inflammation were achieved after statin pre-treatment before renal transplantation [14]. Furthermore, atorvastatin treatment for 10 days significantly reduced infarct volume after cerebral ischemia [15].

Other cardiovascular drugs also possess antioxidant capacity, for example, ACE-inhibitors, calcium antagonists, antiarrhythmic drugs, and *β*-adrenoceptor antagonists [1, 16]. ACE inhibitors have shown to display cardioprotective effects through free radical scavenging [17, 18]. Furthermore, they display anti-inflammatory effects. This is probably achieved by preventing leukocyte adhesion [19]. These beneficial effects were also demonstrated in I/R of rat liver [20]. Calcium antagonists have also demonstrated protective effects in I/R of the liver [21]. All these studies were carried out in animals; little research has been performed in humans in this area.

Administration of statins or other cardiovascular medication would be a safe and simple method to protect patients who are undergoing a medical procedure from the development of I/R damage. The Deep Inferior Epigastric Perforator Free Flap (DIEP) operation is featured by I/R. During this procedure, a tissue flap is dissected from the abdomen and transplanted to the patient's thorax. The tissue flap has to endure around 60 minutes of ischemia. Although this operation is usually successful, in 5–10% of the patients (partial) flap loss occurs. Flap failure after DIEP surgery is a traumatic event for the patient, who already battled against cancer. Flap failure represents another disappointment which leaves the patient with less reconstructive options and the knowledge of having to endure yet another operation. Therefore, much research is being performed in order to prevent the effects of I/R injury. In this study it was examined whether chronic use of statins or other cardiovascular medication protects from the development of I/R related complications after DIEP surgery.

## 2. Materials and Methods

### 2.1. Population

The sample consisted of women that had undergone breast amputation or mastectomy due to breast cancer or in order to prevent breast cancer and underwent breast reconstruction at the Department of Plastic and Reconstructive Surgery in the Maastricht University Medical Centre in The Netherlands between September 2004 and June 2009. Medical information of 134 DIEP patients, amongst whom 39 patients who underwent bilateral surgery, was studied from their medical files.

### 2.2. Medical Information

Of every patient, the medical file was studied for the following patient characteristics: age, length, weight, smoking, and the use of medication. Furthermore, I/R related complications after DIEP surgery were registered (reexploration, necrosis, and (partial) flap loss). Data were processed anonymously and per DIEP flap.

### 2.3. Statistics

With STATA random-effects logistic regression, two statistical models were calculated to answer both research questions. In each statistical model, the patient characteristics age, BMI, and smoking were included and integrated as covariates. For patients who underwent bilateral DIEP surgery, statistical correction was performed. Significance was considered present at *P* ≤ 0.05, two tailed.

## 3. Results

In 13 of the 173 DIEP flaps, statins were used (7.51%) and in 41 flaps other cardiovascular drugs were taken (23.70%). Patient characteristics are shown in Table 1. In 30 Flaps (17.34%) I/R related complications developed.

Statistical analysis showed that the number of I/R related complications after DIEP surgery hadnot decreased due to chronic use of statins. Of the group that did not use statins 16.3% developed complications versus 30.8% amongst patients that did use these drugs (*P* = 0.29). Furthermore, a nonsignificant increasing trend in the occurrence of complications appeared after chronic use of other cardiovascular medication; 14.4% of the patients without medication versus 26.8% of the patients using drugs (*P* = 0.10). Patient characteristics age, BMI, and smoking did not significantly influence the occurrence of complications in both regression models. In 22 out of 173 DIEP flaps, the patient smoked (12.72%).

## 4. Discussion

### 4.1. The DIEP Flap As a Model of I/R

In this study, the DIEP flap was used as a clinical, human model of I/R. It has the advantage of being visible and within reach, even after surgery. Therefore, this new model is well suited for analysing I/R in humans over time. Furthermore, duration of ischemia is relatively constant and patients are healthy, at the time of surgery there is no active disease. Another benefit that the DIEP model offers is that it is an autologous transplantation and there is no interference of donor incompatibility. Therefore, the isolated effect of an intervention can be studied. 

### 4.2. Statins

Statins exert their actions through several mechanisms. Through inhibition of the mevalonate pathway, statins inhibit isoprenoid production [9]. Isoprenoids are responsible for posttranslational modification of many proteins, amongst which is Rho [9]. Rho plays an important role in inflammation by activating transcription factor nuclear factor-kB and it also decreases endothelial production of nitric oxide (NO) [9]. By inhibiting the isoprenylation of Rho and Rho kinase, statins increase eNOS (endothelial nitric oxide synthase) mRNA stability and thereby NO production [3, 4, 7–9]. Statins may also directly activate eNOS through protein kinase Akt activation [8, 12, 13, 22]. Statins activate receptor tyrosine kinases and G-protein-coupled receptors, thereby activating phosphoinositol-3 kinase, which consequently activates the protein kinase Akt by phosphorylation [8]. Next, Akt causes eNOS to be phosphorylated and NO production to increase [9]. Increased availability of NO improves endothelium function and blood flow to the tissues [23].

Secondly, statin administration inhibits upregulation of adhesion molecules, like VCAM-1, ICAM-1 and P-selectin [9, 24]. Hereby, neutrophil rolling, adherence, and neutrophil influx are reduced [25, 26]. This decreased expression of adhesion molecules and PMN infiltration is thought to be regulated through NO release from the endothelium [9, 26–30]. However, how this occurs remains unclear. Some studies demonstrate that eNOS just functions as a trigger for initiating protection, while iNOS (inducible nitric oxide synthase) is the essential mediator in protection through pharmacological preconditioning and is upregulated after statin use [10, 11]. Other studies, on the other hand, show that statins decrease iNOS expression [24, 30].

Research showed that the protective effects of statin treatment could also be mediated by increased prostaglandin production, which is due to an upregulation of cyclooxygenase-2 and other prostaglandin synthases [10]. Cyclooxygenase-2 is the enzyme that catalyses the rate-limiting step in prostaglandin synthesis. Prostaglandins can have beneficial effects during I/R, like anti-inflammatory effects, vasodilation, and platelet disaggregation.

Furthermore, statins display antioxidant effects. They are exerted through many pathways, all resulting in decreased ROS production. First, they inhibit NADPH oxidase, thereby attenuating neutrophil respiratory burst [6]. Furthermore, statins cause S-nitrosylation of thioredoxin, thereby increasing its enzymatic activity and reducing intracellular ROS production [31]. Reduction in ROS production is also achieved by activation of the heme oxygenase-1 promotor in endothelial cells [32]. Heme oxygenases convert heme to biliverdin. Degradation products of heme have the capacity to decrease superoxide anion production [9]. Superoxide production can also be reduced by inhibiting tyrosine phosphorylation in activated neutrophils [33]. Finally, statins downregulate the aldose reductase pathway, which is involved in oxidative stress [14]. Aldose reductase competes with glutathione reductase for NADPH, causing a decrease in reduced glutathione content. Subsequently, the sorbitol metabolism produces NADH, which enables NADH oxidase to produce more ROS [14]. By inhibiting the aldose reductase pathway, statins thus reduce ROS production during I/R and they increase antioxidant capacity by restoring tissue glutathione levels [25].

### 4.3. Cardiovascular Medication

The protective effects of cardiovascular medication are also established through different mechanisms. Calcium antagonists, angiotensin II, and ACE-inhibitors increase blood flow during reperfusion either by vasodilation or through stimulation of angiogenesis [34]. Treatment with angiotensin II and captopril have been demonstrated to stimulate angiogenesis and thereby incline free flap viability and vascularity [35]. Through activation of the AT1 receptor, angiotensin II increases vascular endothelial growth factor expression by vascular smooth muscle and endothelial cells and directly stimulates endothelial cells to produce NO [35].

Furthermore, cardiovascular drugs can prevent ROS formation. First, calcium antagonists inhibit the influx of calcium during I/R [18]. In this process, the lack of ATP leads to ATP-dependent calcium pump dysfunction, causing the intracellular calcium level to increase. This calcium overload triggers conversion of xanthine dehydrogenase to xanthine oxidase, causing the production of ROS [21]. Thus, inhibiting calcium influx prevents ROS formation. *β*-adrenoceptor antagonists also protect from I/R by attenuating calcium influx [16]. Secondly, cardiovascular drugs prevent autoxidation of catecholamines. The antihypertensive effect of ACE-inhibitors is related to inhibition of norepinephrine release from peripheral sympathetic neurons [17]. In the presence of oxygen and transition metals catecholamines could be autoxidized, leading to OH-radical formation [17]. ACE-inhibitors attenuate OH-radical production by decreasing the level of norepinephrine [17]. However, not all ACE inhibitors have radical scavenging properties. It is believed that only ACE inhibitors containing a SH-group in particular possess this capacity [17]. Enalapril, for example, is a non-SH-group containing ACE inhibitor and displayed no protective effects during I/R [18].

The last ROS preventing mechanism is attenuation of neutrophil accumulation during late reperfusion [34]. Just like statins, calcium antagonists can inhibit neutrophil influx, thereby decreasing ROS production from the respiratory burst.

Other working mechanisms of cardiovascular medication do not prevent ROS formation, but increase free radical scavenging properties. Some drugs do this by increasing the antioxidant reserve [18]. Captopril, for instance, increased SOD activity, but induced no changes in glutathione peroxidase and catalase enzyme activity [18]. However, it significantly attenuated lipid peroxidation [18]. Stobadine, a pyridoindole derivative that displays cardioprotective and antiarhythmical effects, showed a protective effect during I/R by increasing the glutathione peroxidase activity and the total antioxidant capacity [36]. ACE inhibitors and calcium antagonists, especially the dihydropyridines, possess radical scavenging properties and prevent ROS formation. They decrease bradykinin degradation, stimulating eNOS to produce NO [19, 34]. NO may act as an antioxidant itself and also prevents activation of polymorphonuclear leukocytes, thereby decreasing the amount of ROS [19]. The increased bradykinin activity stimulates NO and prostacyclin production, causing vasodilation too [18].

### 4.4. Results of This Study

Whether chronic statin treatment offers protective effects in patients undergoing I/R-during surgical procedures, remains unclear. Although some studies demonstrated a decrease in C-reactive protein, plasma adhesion molecule levels and cytokine levels [29, 37–40], other studies could not confirm these findings [41, 42]. And whether these changes result in a clinical benefit, like a decrease in I/R related complications, is not clear either. Pascual et al. demonstrated a decrease in early complications after coronary artery bypass grafting, but these beneficial effects only occurred in patients with a positive troponin T status [41]. This beneficial effect might be caused by an effect on atherosclerotic plaques or cholesterol level rather than a general antioxidant or anti-inflammatory mechanism. Patti et al. and the study of Pasceri and colleagues both demonstrated a decrease in postprocedural complications after statin use [42, 43]. However, this was achieved after short-term pre-treatment (7 days). An animal study showed that short-term statin administration could have protective effects, while these effects are absent after chronic statin treatment [44].

There is little evidence proving a clinical protective effect of chronic statin use. Our study could not demonstrate a beneficial effect either. Chronic use of statins did not decrease the occurrence of I/R related complications after DIEP surgery. Unfortunately, the group that endured statin pre-treatment was small (13 flaps). There are studies demonstrating evidence that *short-term* pre-treatment with statins could be effective in preventing I/R injury. Possibly the body adapts to chronic statin use, thereby compensating the beneficial effects of statins on I/R. Therefore, further research has to be aimed at evaluating the effects of short-term pre-treatment with statins before I/R.

The use of cardiovascular medication even showed a mild increase in the number of complications. In this category different types of drugs have been included. The effects of these drugs on the process of I/R are different. Some drugs might not have a protective effect at all, like non-SH-group containing ACE inhibitors. It is possible that a study in which the different types of cardiovascular drugs are studied separately, shows a different result. However, in our study no distinction was made because groups would be small due to the large variety of cardiovascular medications and because many patients use multiple types of drugs.

Furthermore, patients in this study that use statins or other cardiovascular drugs do this based on a medical indication. This means all patients in the statin and cardiovascular group suffer from hypertension or dyslipidemia, no patients displayed cardiovascular disease. Probably, the decreased vascular condition and perfusion of these patients is the reason for the increased risk of complications. The beneficial effects of these drugs as described in the literature are probably occurring, but are not strong enough to compensate for the deleterious vascular condition of these DIEP patients. The same accounts for the statin group in our study.

We had the idea to study whether a beneficial effect of chronic use of statins or other cardiovascular medication could be demonstrated from a medical file study in DIEP patients, but unfortunately this was not the case. The main disadvantage of this study is its observational, retrospective design. However, it demonstrated the probability that chronic treatment with statins and other cardiovascular medication influences the occurrence of I/R related complications and thereby gave important directions for further research. Because there is ample literature demonstrating evidence that *short-term* pre-treatment with statins could be effective in preventing I/R injury, future research should be aimed at short-term pre-treatment instead of chronic use. Ideally, this would be performed as an intervention study in which these drugs are administered for a short period to healthy patients undergoing I/R.

In conclusion, this study failed to demonstrate a protective effect from the chronic use of statins or other cardiovascular medication on the effects of I/R. However, there is evidence demonstrating beneficial effects from short-term pre-treatment with statins. Further research in this area should therefore be focussed on short-term premedication with statins. The most important feature in this study is the use of the DIEP operation as a human model of I/R. In contrast to other models, this new, clinical model is well suited for analysing I/R in humans over time because the DIEP flap remains within reach after surgery and interference of donor incompatibility is avoided.

## Figures and Tables

**Table 1:** patient characteristics per study group.

	All	Statin	Cardiovascular
*N* (flaps)	173	13	41
Age (yr)	47.67	58.31	53.93
BMI (kg^2^/m^2^)	27.17	28.40	28.60
Smoking (%)	12.72	0	2.44
Complication (%)	17.34	30.77	26.83
